# In vitro proof of concept studies of radiotoxicity from Auger electron-emitter thallium-201

**DOI:** 10.1186/s13550-021-00802-w

**Published:** 2021-07-05

**Authors:** Katarzyna M. Osytek, Philip J. Blower, Ines M. Costa, Gareth E. Smith, Vincenzo Abbate, Samantha Y. A. Terry

**Affiliations:** 1 School of Biomedical Engineering and Imaging Sciences, King’s College London, London, UK; 2 Theragnostics Limited, 2 Arlington Square, Bracknell, RG12 1WA UK; 3 Department of Analytical, Environmental and Forensic Sciences, King’s College London, London, UK

**Keywords:** Auger electrons, ^201^Tl, Thallium-201, Radiobiology, Targeted molecular radionuclide therapy

## Abstract

**Background:**

Auger electron-emitting radionuclides have potential in targeted treatment of small tumors. Thallium-201 (^201^Tl), a gamma-emitting radionuclide used in myocardial perfusion scintigraphy, decays by electron capture, releasing around 37 Auger and Coster–Kronig electrons per decay. However, its therapeutic and toxic effects in cancer cells remain largely unexplored. Here, we assess ^201^Tl in vitro kinetics, radiotoxicity and potential for targeted molecular radionuclide therapy, and aim to test the hypothesis that ^201^Tl is radiotoxic only when internalized.

**Methods:**

Breast cancer MDA-MB-231 and prostate cancer DU145 cells were incubated with 200–8000 kBq/mL [^201^Tl]TlCl. Potassium concentration varied between 0 and 25 mM to modulate cellular uptake of ^201^Tl. Cell uptake and efflux rates of ^201^Tl were measured by gamma counting. Clonogenic assays were used to assess cell survival after 90 min incubation with ^201^Tl. Nuclear DNA damage was measured with γH2AX fluorescence imaging. Controls included untreated cells and cells treated with decayed [^201^Tl]TlCl.

**Results:**

^201^Tl uptake in both cell lines reached equilibrium within 90 min and washed out exponentially (*t*_1/2_ 15 min) after the radioactive medium was exchanged for fresh medium. Cellular uptake of ^201^Tl in DU145 cells ranged between 1.6 (25 mM potassium) and 25.9% (0 mM potassium). Colony formation by both cell lines decreased significantly as ^201^Tl activity in cells increased, whereas ^201^Tl excluded from cells by use of high potassium buffer caused no significant toxicity. Non-radioactive TlCl at comparable concentrations caused no toxicity. An estimated average ^201^Tl intracellular activity of 0.29 Bq/cell (DU145 cells) and 0.18 Bq/cell (MDA-MB-231 cells) during 90 min exposure time caused 90% reduction in clonogenicity. ^201^Tl at these levels caused on average 3.5–4.6 times more DNA damage per nucleus than control treatments.

**Conclusions:**

^201^Tl reduces clonogenic survival and increases nuclear DNA damage only when internalized. These findings justify further development and evaluation of ^201^Tl therapeutic radiopharmaceuticals.

**Supplementary Information:**

The online version contains supplementary material available at 10.1186/s13550-021-00802-w.

## Background

Thallium-201 (^201^Tl, *t*_1/2_ = 73 h) is well-known in diagnostic nuclear medicine. By mimicking the biological behavior of potassium, it has played a major role in myocardial perfusion scintigraphy [1], until it was largely replaced by ^99m^Tc labeled complexes [2]. ^201^Tl has also been used for tumor imaging [3, 4], to evaluate chemotherapy responses [5] and tumor malignancy [6, 7]. However, because of low signal-to-noise ratio, significant tissue attenuation and high radiation absorbed dose [8], diagnostic use of ^201^Tl has substantially diminished in recent years.

Apart from its gamma rays (135 keV—3%, 167 keV—10%) and X-rays (67–83 keV—94%) used in clinical imaging [9], ^201^Tl decay by electron capture to stable mercury (^201^Hg) also produces around 37 short-range Auger and Coster–Kronig electrons, one of the highest numbers among other Auger-electron emitters (Table 1) [10] with therapeutic potential. The average total energy of Auger and Coster–Kronig electrons per decay is calculated as 15.3 keV [10] and even though this is comparable to the energy of ^125^I, its half-life is around 20 times shorter and hence more amenable to clinical use. Auger electron-emitters such as ^111^In,^125^I and ^67^Ga (Table 1) [10], have already shown potential for targeted radionuclide therapy of cancer [11–13], especially when delivered close to sensitive cellular targets such as DNA and plasma membrane [10, 14, 15]. However, the therapeutic potential of ^201^Tl has barely been studied, despite its potent shower of Auger electrons and good worldwide availability. Nearly 40 years ago, in the context of the safety of diagnostic use of ^201^Tl, Rao et al. observed radiotoxic effects of ^201^Tl in spermatogonial cells after intratesticular injection in a mouse model [16], and Kassis et al. compared the cellular kinetics and radiotoxicity of ^201^Tl to those of β-emitters (^42^K, ^43^K and ^86^Rb) in Chinese hamster lung fibroblasts [17]. Since then there has been little interest in ^201^Tl radiobiology or radionuclide therapy.

**Table 1:** Characteristics of some of the Auger electron emitters [10]

	^201^Tl	^111^In	^67^Ga	^125^I
Half-life (days)	3.04	2.80	3.26	59.4
Number of Auger and Coster–Kronig electrons /decay	36.9	14.7	4.7	24.9
Auger and Coster–Kronig electrons energy per decay (keV)	15.3	6.8	6.3	12.2
Associated gamma emission (keV)	135.3; 167.4	171.3; 245.4	9.1; 9.3; 184; 209; 300; 393	3535

**Fig. 1:**
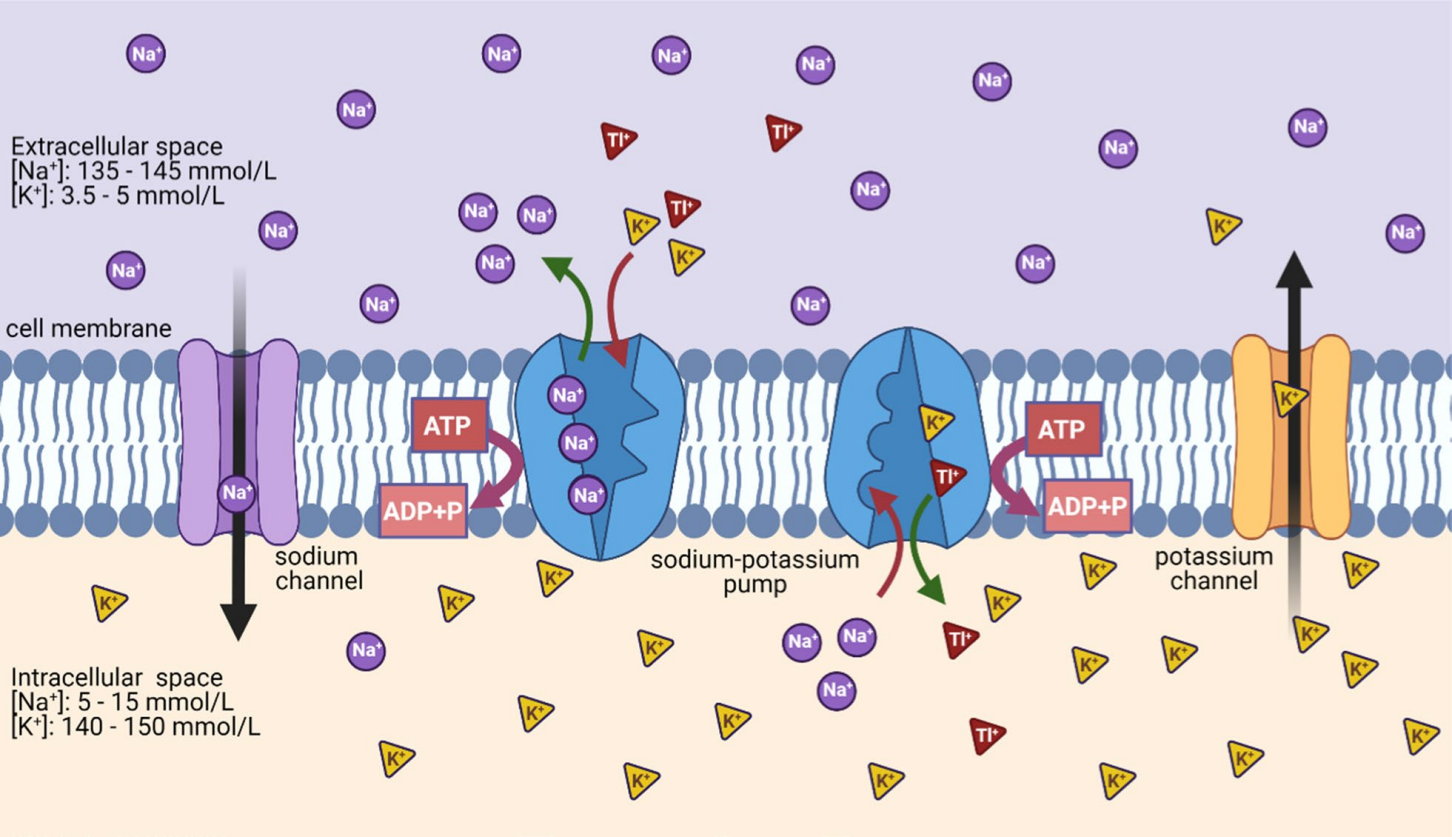
Schematic representation of K^+^, Na^+^ and Tl^+^ transport across cellular membranes. Sodium–potassium pump (Na^+^/K^+^-ATPase) transports K^+^ ions inside and Na^+^ ions outside a cell against their concentration gradient using energy released by the hydrolysis of ATP. Tl^+^ ions, which mimic K^+^ physiological behaviour, can be transported inside the cell via potassium channels, such as the sodium–potassium pump. Tl^+^ transport mechanism outside the cell is currently unknown

In this preliminary evaluation of the potential of ^201^Tl as a therapeutic radionuclide, we investigated the rate and extent of non-targeted ^201^Tl uptake into cancer cells via potassium channels, such as the Na^+^/K^+^ pump (Fig. 1), and used this mechanism of accumulation to assess its radiotoxic effect in human cancer cells in vitro*.* The primary aim of this study was to measure the effect of internalized and non-internalized ^201^Tl on clonogenic survival and DNA integrity in prostate and breast cancer cells. Non-targeted ^201^TlCl was used in this study only to assess the radiotoxic potential of the radionuclide, and not to assess the potential of ^201^TlCl itself. In contrast to other Auger electron-emitting electrons, such as ^111^In or ^67^Ga, no suitable chelators are available for ^201^Tl and their development can be challenging due to its physical and chemical properties. A finding that ^201^Tl has potential as a therapeutic radionuclide would stimulate development of effective thallium chelators for construction of targeted bioconjugates.

## Methods

Cell culture consumables, unless specified, were purchased from Sigma-Aldrich, UK.

[^201^Tl]TlCl in sterile 0.9% NaCl solution (360–560 MBq/5.8 mL) was obtained from Curium Pharma, UK; [^201^Tl]TlCl specific radioactivity was greater than or equal to 3.7 MBq/μg of thallium and radiochemical purity was at least equal to 95% (as per [^201^Tl]TlCl specification). [^201^Tl]TlCl was used in all experiments without any preconditioning.

### Cell culture

Human breast adenocarcinoma cells MDA-MB-231 (ATCC® HTB-26™) were cultured in Dulbecco’s Modified Eagle Medium (DMEM, low glucose 1000 mg/L) and human prostate carcinoma cells DU145 (ATCC® HTB-81™) in RPMI-1640 medium. Both media were supplemented with 10% fetal bovine serum, 5% L-glutamine, penicillin (100 units) and 100 μg/mL streptomycin. Cultured cells were trypsinized and seeded at 250,000 cells per well in 24-well plates 16 h before each experiment and grown at 37 °C in a humidified 5% CO_2_ atmosphere. During experiments, cells were kept in full medium or phosphate buffered saline (PBS; D8537) supplemented with MgCl_2_ and CaCl_2_ in concentrations equivalent to those found in media, or PBS without potassium (Alfa Aesar, J60465) supplemented as described above for PBS. KCl solutions were prepared by dissolving KCl (BDH Laboratory) in 0.9% NaCl. Concentrations of K^+^ and Na^+^ in PBS, PBS without K^+^, RPMI and DMEM media are shown in Table 2.

**Table 2:** Concentration of potassium and sodium in incubation solutions used in experiments

Incubation solution	Concentration of K^+^ (mmol/L)	Concentration of Na^+^ (mmol/L)
Dulbecco’s Modified Eagle Medium (DMEM)	5.3	155.0
RPMI-1640 medium	5.3	138.5
Phosphate buffer saline (PBS)	4.2	153.0
Phosphate buffer saline (PBS) without K^+^	0	157.0

Concentration of potassium and sodium in incubation solutions used in experiments. To modulate ^201^Tl uptake in cells, 0 mmol/L K^+^ solutions were supplemented with KCl at a range of concentrations up to 25 mmol/L

### Uptake and efflux assays

Fifteen minutes before each experiment the medium in each well was replaced by 200 µL fresh medium, PBS or PBS without K^+^. Stock [^201^Tl]TlCl was diluted with 0.9% NaCl to the required concentration (1–40 MBq/mL) and 50 µL (50–2000 kBq) was added to each well. Plates were incubated at 37 °C for a specified period. Then, the radioactive incubation solution was collected. Adherent cells were briefly washed thrice with PBS and lysed with 1 M NaOH for 15 min at room temperature (RT). Unbound radioactivity (incubation medium and PBS washings) and cell-bound radioactivity (lysate) were measured (counts per minute, CPM) with a CompuGamma CS1282 gamma counter.

To measure the rate of ^201^Tl washout, cells were incubated at 37 °C for 90 min with 50 kBq (1 MBq/mL) ^201^Tl in 250 µL medium (total), which was then removed. Adherent cells were washed briefly with 250 µL PBS and 250 µL of fresh non-radioactive medium was added to each well. Cells were incubated for various times (from 15 to 180 min) at 37 °C, after which medium was collected and cells washed and lysed as described above. A second method, to assess continuous complete ^201^Tl efflux, involved repeated medium changes on the same cells. In brief, after incubation with 50 kBq (1 MBq/mL) ^201^Tl as above, replacing radioactive medium with fresh medium and allowing a further 15 min of efflux; medium was again removed, cells were washed once with PBS and fresh medium added. This process was repeated 4 times at intervals from 15 to 60 min. After 180 min cells were lysed and ^201^Tl activity measured as described above, converting CPM to activity by means of a calibration curve (Additional file 1: Fig. S1). A nominal average intracellular volume of 1.6 pL for individual DU145 cells (as measured by diffusion NMR spectroscopy [18]) was used for intracellular-to-extracellular concentration ratio calculations.

^203^Tl is a stable isotope used as a target material in the ^201^Tl manufacturing process [19] and despite a rigorous purification process, some ^203^Tl is still present in [^201^Tl]TlCl solution (Additional file 1: Fig. S2 and Table S1). Inductively Coupled Plasma-Mass Spectrometry (ICP-MS) analysis was performed using a PerkinElmer NexION350D ICP-Mass Spectrometer to estimate ^203^Tl concentrations in [^201^Tl]TlCl decayed samples, using a calibration curve prepared as described in Additional file 1.

### DNA damage

γH2AX assays were used to quantify DNA double-strand breaks (DSBs). Cells were seeded on coverslips coated with poly-L-lysine (50 µg/mL) placed in each well of a 24-well plate. Following a 90-min incubation with 50 µL (50–2000 kBq) of different ^201^Tl activities (200–8000 kBq/mL), ^203^Tl/^201^Hg in equivalent concentrations, or ^201^Tl (1000 kBq, 4000 kBq/mL) with added KCl (15 and 25 mM), medium was removed and coverslips were washed with PBS, fixed with 3.7% formalin in PBS, treated for 15 min with 0.5% Triton X-100® and 0.5% IGEPAL CA-630® solution, incubated with 1% goat serum/2% bovine serum albumin (BSA) in PBS for 1 h, washed with PBS, incubated overnight at 4 °C with mouse antiphospho-histone H2A.X monoclonal antibody (Merck, 1:1600 in 3% BSA), washed with 3% BSA in PBS and incubated with goat anti-mouse secondary fluorescent antibody Alexa Fluor®488 (Invitrogen, 1:500 in PBS) for 2 h at 4 °C. Hoechst 33,342 nuclei stain was added for 2 min at RT. Coverslips were washed twice with PBS and attached to microscope slides using 2.5% DABCO/Mowiol® (except for the experiment blocking ^201^Tl uptake with KCl, where cells were stained and mounted with Prolong™ Gold Antifade Reagent with DAPI (Invitrogen)). Slides were stored at 4 °C. A TCS SP5 confocal microscope with Leica software was used to obtain fluorescent images and CellProfiler was used to quantify foci numbers per nucleus.

### Cytotoxicity

^201^Tl (50–2000 kBq, 200–8000 kBq/mL), ^203^Tl/^201^Hg in equivalent concentrations, or ^201^Tl (1000 kBq, 4000 kBq/mL) with KCl (15 and 25 mM) was added to MDA-MB-231 or DU145 cells in 200 µL medium or potassium-free PBS. After 90 min, the radioactive incubation solution was removed, cells were washed three times with PBS, trypsinized, re-suspended in non-radioactive medium, seeded at 1000 cells/well in a 6-well plate and cultured for 8–10 days, changing medium every 2–3 days. Colonies were fixed and stained with 0.05% crystal violet in 50% methanol and counted manually with blinding, defining colonies as containing > 50 cells. All clonogenic assays were done alongside uptake assays to relate activity per cell with the surviving fraction.

### Statistical analysis

Data were analysed in Excel Microsoft 2016 and GraphPad Prism 8, and expressed as mean ± SD. A nonparametric Mann–Whitney test for unpaired data was used to determine significance (*P* < 0.05). Each experiment was performed in triplicate or quadruplicate. Error bars represent standard deviations among independent experiments, except when *n* = 1 where the error is intra-experimental.

The intracellular activity was calculated comparing the gamma counter measurements (cpm) of the collected medium activity (with three PBS washes) to the lysate (NaOH) activity. The background count (BC) was subtracted from both values.

$${\text{Intracellular\,activity}}\,(\%) = \frac{{{\text{lysate~activity}} - {\text{BC}}}}{{\left( {{\text{lysate~activity}} - {\text{BC}}} \right) + \left( {{\text{collected~medium~activity}} - {\text{BC}}} \right){\text{~}}}}~*100$$

For the gamma counter measurements, fractions of the culture medium and lysate were taken (when needed) and then multiplied by the appropriate factor to obtain total values necessary for calculations.

The intracellular activity per cell was calculated by multiplying the activity added to each well (50–2000 kBq) by the measured intracellular activity (%) and divided by the number of cells per well counted right after the experiment.

$${\text{Intracellular\,activity\,per\,cell}}\,({\text{Bq}}/{\text{cell}}) = \frac{{{\text{activity}}\,{\text{added\,per\,well}}\,({{\text{Bq}}})*{\text{intracellular}}\,{\text{activity}}\,(\%)}}{{{\text{number\,of\,cells\,per\,well}}*100}}$$

Figures were created with GraphPad Prism 8 and http://www.BioRender.com.

## Results

### ^201^Tl kinetics: uptake and efflux

**Fig. 2:**
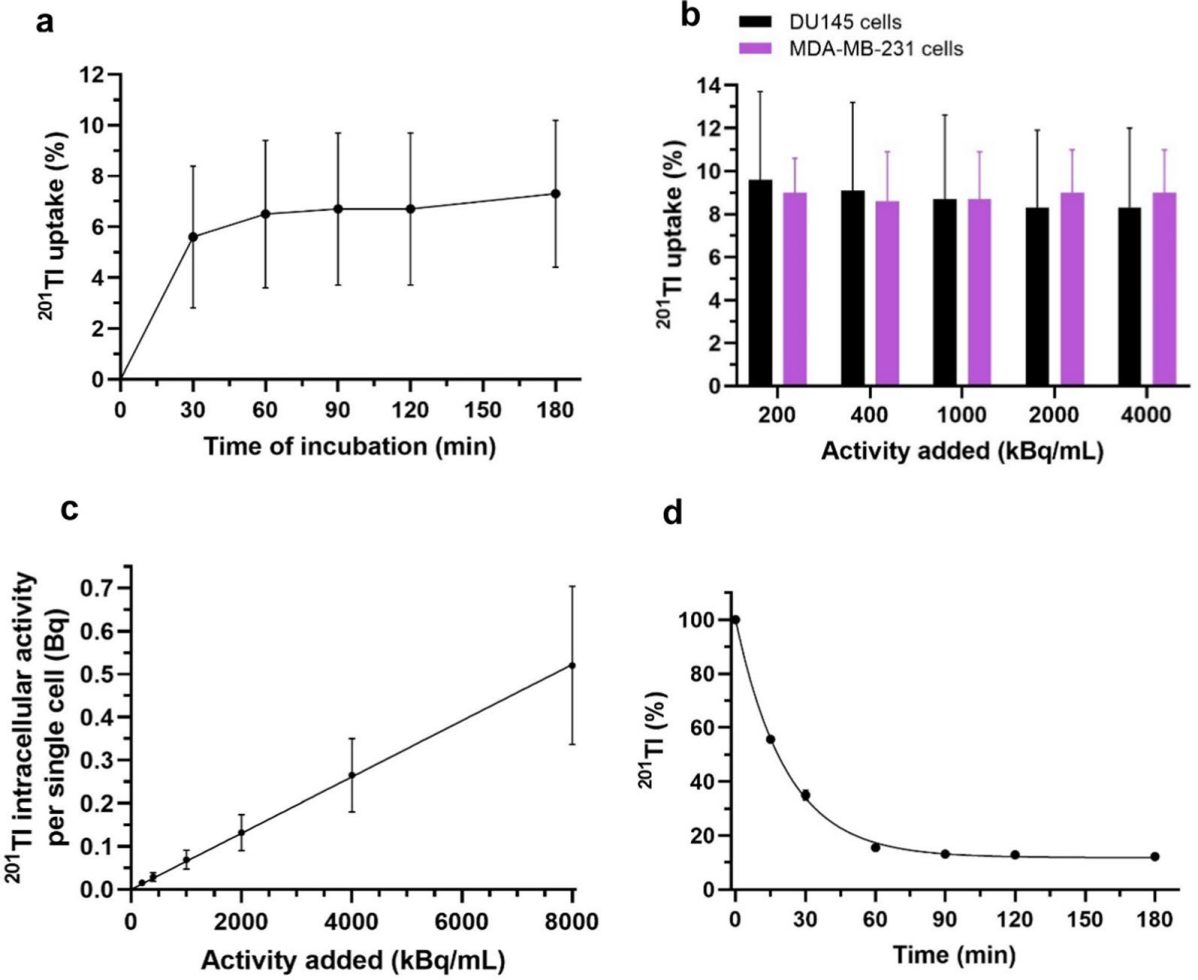
^201^Tl kinetics. **a** Uptake of ^201^Tl in DU145 cells (*n* = 3) after incubation with 200 kBq/mL [^201^Tl]TlCl in medium. These results were used to determine the best incubation time for subsequent radiobiological experiments. **b** ^201^Tl uptake in DU145 cells (*n* = 3) and MDA-MD-231 cells (*n* = 2) after 90 min incubation in medium with different activities of [^201^Tl]TlCl. **c** ^201^Tl intracellular activity per single cell (Bq) versus activity added (kBq/mL) in DU145 cells (*n* = 3) measured in medium. Graphs b and c show that the increasing activity of ^201^Tl does not inhibit its cellular uptake, suggesting that saturation of uptake mechanisms is not reached at the activities used in this work. **d** Efflux of ^201^Tl in DU145 (*n* = 3) cells. Plateau uptake from the preceding uptake assay is defined as 100% at time 0. Radioactive medium was replaced with non-radioactive and ^201^Tl activity was measured over time. Data are presented as mean ± SD, done in triplicates; exponential regression line was fitted in d. Some error bars are smaller than the data symbols and hence not visible

In preliminary experiments in RPMI or DMEM medium (Table 2), cellular uptake of ^201^Tl in both DU145 and MDA-MB-231 cells rose sharply up to 60 min and then plateaued (Fig. 2a and Additional file 1: Fig. S3a). The % uptake was independent of the activity used within the range examined (Fig. 2b, c) showing that the uptake mechanism was not saturable in this range. Based on these results: 90 min incubation time was chosen for radiobiological experiments. Efflux rate measurements were performed after 90 min ‘loading’ with 200 kBq/mL of ^201^Tl in RPMI or DMEM medium. In both cell lines, when the radioactive medium was replaced by non-radioactive medium, ^201^Tl bound by cells decreased exponentially with a half-life of around 15 min reaching a new equilibrium (Fig. 2d and Additional file 1: Fig. S3b) with the same intracellular-to-extracellular ratio of ^201^Tl concentration. When medium was repeatedly refreshed, we again observed an exponential drop in the activity bound per cell, from 0.017 to 0.019 Bq at the beginning of the efflux experiment to approach 0 after 180 min (Additional file 1: Fig. S3c).

To modulate ^201^Tl uptake into cells, cardiac glycosides digoxin and ouabain were evaluated but rejected because of their toxic effects on cells as described in Supplementary Material (Additional file 1: Fig. S4a and b) [20]. Instead, the effect of varying potassium concentration was evaluated. DU145 cells were incubated with 80 kBq/mL of ^201^Tl for 90 min in PBS solution without potassium (Table 2) and supplemented with different concentrations of KCl rising to 50 mM. ^201^Tl uptake decreased from 23.6 ± 0.6% in 0 mM potassium to 1.1 ± 0.1% in 50 mM potassium (Additional file 1: Fig. S4c). The potassium concentrations used showed no measurable baseline effects as measured by clonogenic assay or yH2AX imaging (see below), therefore variation of potassium concentration was adopted as the method of choice to modulate ^201^Tl uptake.

### ^201^Tl-induced nuclear DNA damage (yH2AX assay)

**Fig. 3:**
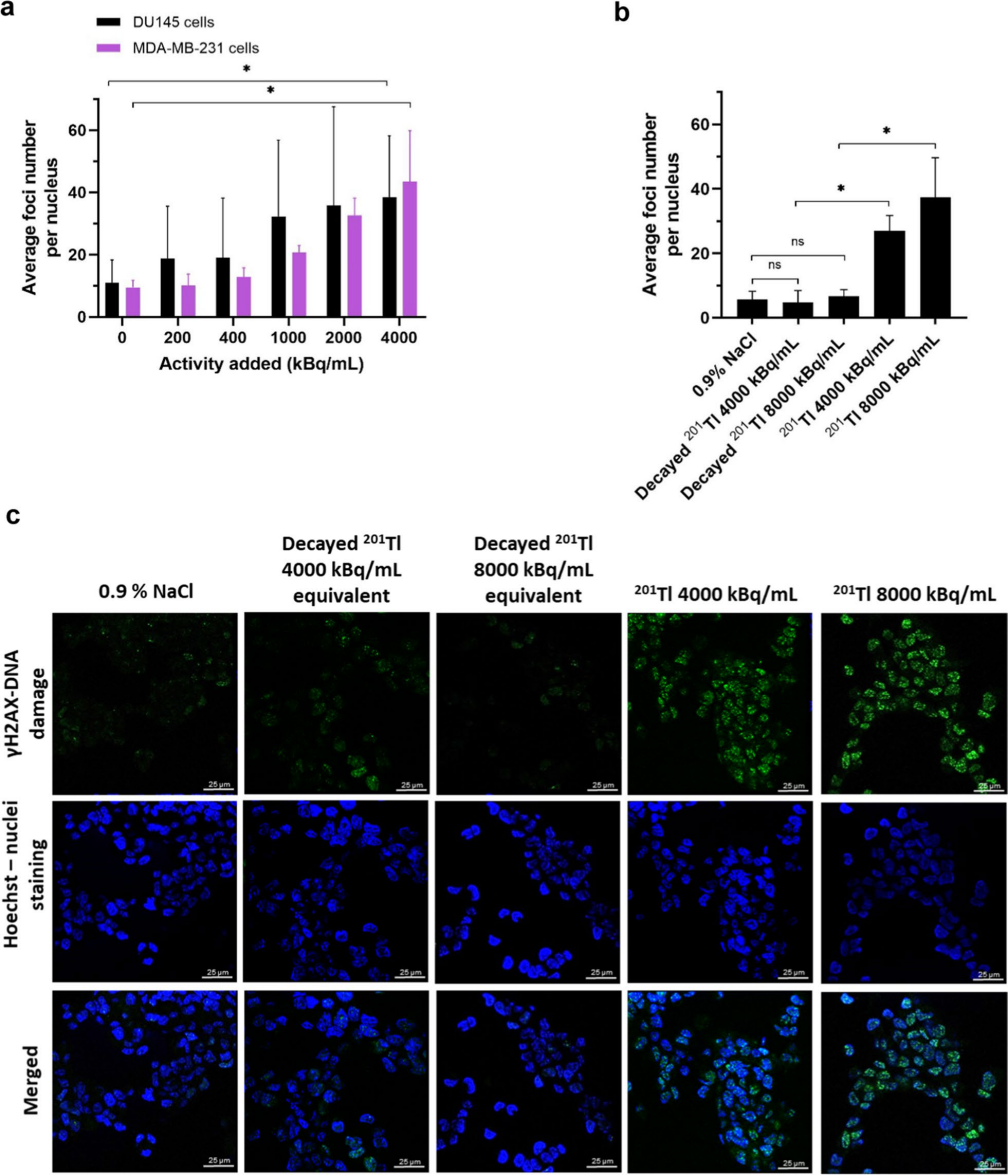
^201^Tl radiotoxicity—nuclear DNA damage. Average number of foci per nucleus in DU145 cells and MDA-MD-231 cells after 90 min incubation **a** with [^201^Tl]TlCl in medium **b** with 0.9% NaCl (negative control), an equivalent volume of [^201^Tl]TlCl-decayed sample or [^201^Tl]TlCl. A range of 26–72 nuclei per condition were analysed across all experiments; same settings were kept within the experiment. 3–9 pixel units were set as a typical diameter of foci. Bars represent mean ± SD, *n* = 3, * indicates *P* < 0.05, ns—not significant. **c** Exemplar confocal microscopy images (100×, field contain minimum 26 nuclei) of DU145 cells incubated for 90 min with 0.9% NaCl (negative control), an equivalent volume of [^201^Tl]TlCl-decayed sample or [^201^Tl]TlCl followed by immunofluorescence staining with green fluorescence for γH2AX (smart gain and smart offset were kept constant for all conditions in the experiment). Nuclear DNA is stained with Hoechst (blue). Scale bar—25 μm

Results from three experiments on DU145 and MDA-MB-231 cells are shown in (Fig. 3a, Table 3). Cells treated with 4000 kBq/mL of ^201^Tl showed a significantly higher average number of foci per nucleus (3.5 and 4.6 times for DU145 and MDA-MB-231 cells, respectively) than in the 0.9% NaCl-treated control (*P* < 0.05, Mann–Whitney test) (Additional file 1: Fig. S5a and b). Comparing the nuclear DNA damage in DU145 cells caused by 4000–8000 kBq/mL of ^201^Tl to that caused by an equivalent volume of decayed [^201^Tl]TlCl sample, we observed that only the radioactive component of the samples significantly increased the average number of foci per nucleus (*P* < 0.05, Mann–Whitney test) (Fig. 3b). The decayed sample caused no visible DNA damage compared to untreated controls (Fig. 3c) as expected, since thallium concentration is very low and other heavy metals (such as Hg decay product) were not detectable by ICP-MS (see Additional file 1: Fig. S2a and b and Table S1).

**Table 3.** ^201^Tl radiotoxicity—nuclear DNA damage

Activity of ^201^Tl (kBq/mL)	Average number of foci per nucleus (± SD) DU145			Average number of foci per nucleus (± SD) MDA-MB-231		
0	3.5 ± 3.6	17.8 ± 18.3	11.9 ± 10.4	7.9 ± 10.3	8.6 ± 6.2	12.0 ± 10.9
100	5.2 ± 5	37.5 ± 25.2	13.3 ± 10.6	7.0 ± 5.3	9.4 ± 7.5	14.0 ± 13.1
200	4.4 ± 4.1	40.6 ± 33.7	12.0 ± 9.1	13.8 ± 8.0	9.5 ± 5.3	15.0 ± 7.2
400	16.2 ± 11.5	60.4 ± 34.9	19.9 ± 12.8	18.2 ± 9.4	22.7 ± 11.0	21.0 ± 12.2
1000	10.1 ± 6.2	71.2 ± 23.2	26.2 ± 18.1	30.9 ± 18.0	27.9 ± 17	38.8 ± 20.4
4000	28.6 ± 14.7	61.2 ± 27.8	25.3 ± 13.0	61.5 ± 28.0	29.6 ± 12.7	39.2 ± 21.5

Average number of foci per nucleus in DU145 cells and MDA-MD-231 cells after 90 min incubation with various activities of [^201^Tl]TlCl in medium**,** and with 0.9% NaCl (0 kBq/mL) measured in three independent experiments

### ^201^Tl radiotoxicity: clonogenic survival

**Fig. 4:**
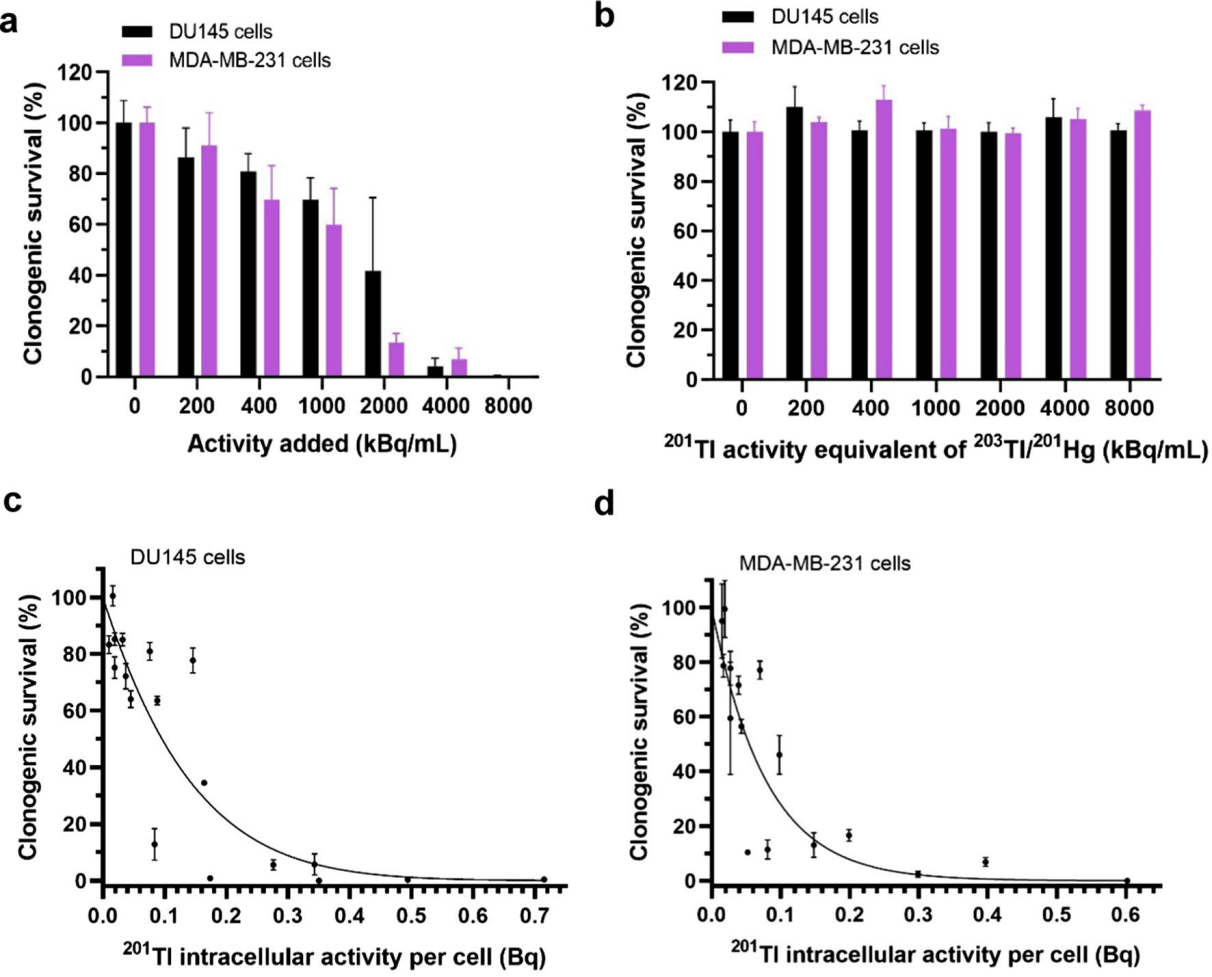
^201^Tl radiotoxicity—clonogenic survival. **a** Clonogenic survival (%) in DU145 (*n* = 3) and MDA-MD-231 cells (*n* = 3 except 8000 kBq/mL where *n* = 1) after 90 min incubation with [^201^Tl]TlCl in medium. **b** Clonogenic survival (%) measured in DU145 cells (*n* = 3) and MDA-MB-231 cells (*n* = 1) after 90 min incubation in medium with different concentrations of non-radioactive [^201^Tl]TlCl decayed sample. **c** Clonogenic survivals (%) versus ^201^Tl intracellular activity per single cell in DU145 cells (*n* = 3) and **d** MDA-MB-231 cells (*n* = 3). Linear quadratic survival model was fitted and calculated activity causing at least 90% reduction was 0.29 Bq for DU145 cells (95% CI 0.18–0.49) and 0.18 Bq for MDA-MB-231 (95% CI 0.10–0.39). Some error bars are smaller than the data points and not visible. Data are presented as mean ± SD, triplicates

Figure 4a presents clonogenic survival results for both cell lines incubated with various activities of ^201^Tl for 90 min in RPMI or DMEM medium ([K^+^] 5.3 mM). No reduction in surviving fractions was observed in cells treated with an equivalent volume of the decayed ^201^Tl sample in DU145 cells (Fig. 4b). An estimated average ^201^Tl intracellular activity per cell of 0.29 Bq (95% CI 0.18–0.49 Bq) for DU145 cells and 0.18 Bq (95% CI 0.10–0.39 Bq) for MDA-MB-231 cells (Fig. 4c, d) reduced clonogenic survival by at least 90% compared to untreated cells.

### The impact of ^201^Tl internalisation on clonogenic survival and nuclear DNA damage in DU145 cells

**Fig. 5:**
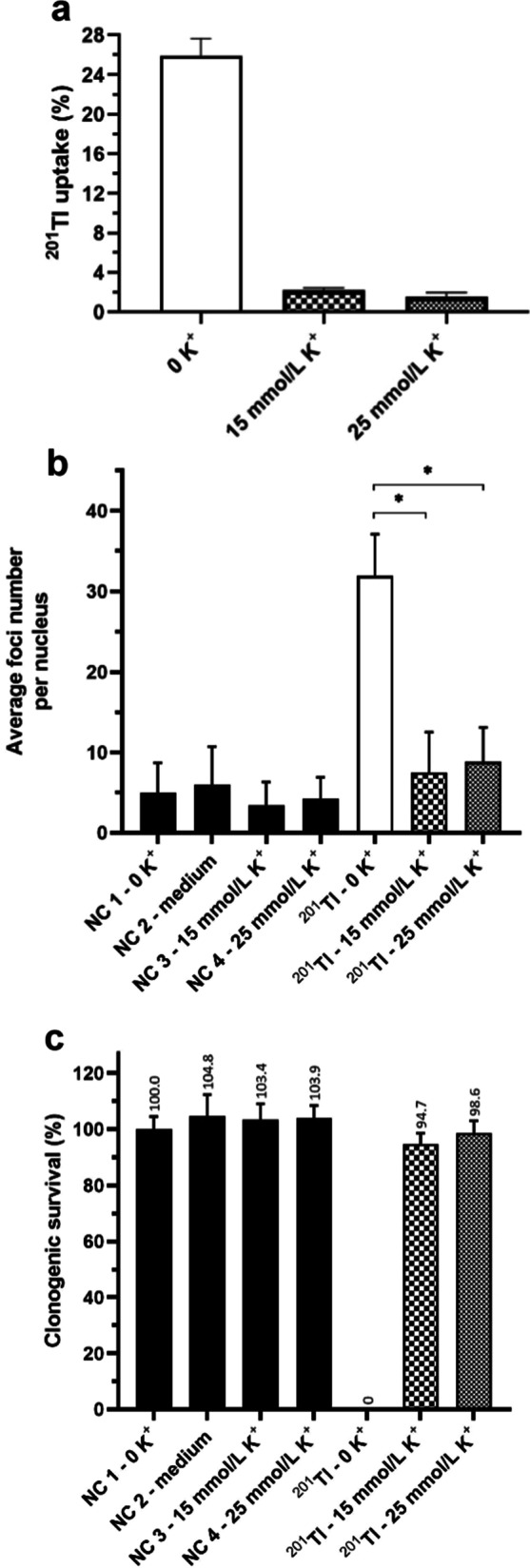
^201^Tl radiotoxicity—internal vs external ^201^Tl. **a** ^201^Tl uptake in DU145 cells in PBS with different concentrations of K^+^ (0–25 mM) after 90 min incubation with 4000 kBq/mL of [^201^Tl]TlCl. **b** Average number of foci per nucleus and **c** clonogenic survival measured in DU145 cells after 90 min incubation with 4000 kBq/mL of [^201^Tl]TlCl and 15 or 25 mmol/L KCl and various non-radioactive controls (NC1-4). Data are presented as mean ± SD, *n* = 3, triplicates, * indicates *P* < 0.05

Varying potassium concentrations in the incubation solution above and below the physiological level (3.5–5 mM [21]) was adopted as a method to modulate ^201^Tl uptake in cells, for the purpose of comparing the radiotoxicity of intracellular and extracellular ^201^Tl. Applying this method, we used the yH2AX assay to compare the average number of foci per nucleus in DU145 cells with high ^201^Tl uptake ([K^+^] 0 mM, 25.9 ± 1.7% uptake, 181:1 intra:extra ratio) and low ^201^Tl uptake ([K^+^] 15 and 25 mM, 1.6 ± 0.4 and 2.2 ± 0.2% uptake respectively, 8:1 and 12:1 intra:extra ratio) after incubation with the same radioactive concentration (4,000 kBq/mL) of ^201^Tl (Fig. 5a, b) and (Additional file 1: Table S2). We observed a significant reduction in the average number of foci per nucleus induced by ^201^Tl—from 31.9 ± 5.2 at 0 mM K^+^ to 8.8 ± 4.3 and 7.5 ± 5.0 at 15 and 25 mM potassium respectively (*P* < 0.05, Mann–Whitney test). In cells incubated with these K^+^ concentrations without ^201^Tl or non-radioactive Tl (NC1,3–4), there was no increase in foci per nucleus compared to cells incubated in standard RPMI medium ([K^+^] 5.3 mM) (NC2). These results show that ^201^Tl only induced DNA DSBs when internalized; extracellular ^201^Tl showed no effect. A similar result emerged from clonogenic survival assays. Incubation of DU145 cells for 90 min with 4000 kBq/mL of ^201^Tl (Fig. 5c) was lethal (no clonogenic survival) in 0 mM potassium medium, but 94.7 ± 3.9 to 98.6 ± 4.4% survival was found when ^201^Tl uptake was suppressed with 15 or 25 mM KCl. Both extremes of potassium concentration showed no clonogenic toxicity in the absence of ^201^Tl. Thus, preventing or reducing cellular uptake of the ^201^Tl by increasing potassium concentration in the medium prevented both the DNA damage and clonogenic toxicity of ^201^Tl.

## Discussion

^201^Tl is efficiently taken up by MDA-MB-231 cells and DU145 cells in standard incubation media ([K^+^] 4.2–5.3 mM), achieving equilibrium at around 90 min with an estimated intracellular to extracellular ^201^Tl concentration ratio (in DU145 cells) in the range 40:1–53:1 (Additional file 1: Table S2a and b) (very similar to literature estimates for K^+^ ratios of 30:1–50:1 [16]). This ratio rises to 181:1 in potassium-free medium and collapses to 8:1 at elevated potassium levels (25 mM) (Additional file 1: Table S2c). These findings are consistent with the assumption that Tl^+^ mimics potassium in a biological environment. ^201^Tl efflux results showed exponential time-dependent efflux when the incubation medium was replaced by ^201^Tl-free medium, returning to a new equilibrium with similar internal to external concentration ratio, suggesting that there is no significant irreversible binding mechanism for thallium ions inside cells in the short time scale of these experiments. The two efflux methods used confirmed that the chemical form of thallium is unchanged after entering the cell and the uptake mechanism is completely reversible, since the same ratios are preserved at every wash; and that only by the continuous efflux method can radioactivity be completely removed from the cells. Nevertheless, the high intracellular to extracellular concentration ratio achievable allowed evaluation of radiotoxicity of internalized ^201^Tl without having a specific tumor-targeted uptake mechanism.

The radiotoxic effect of ^201^Tl uptake in both cell lines was assessed by measuring clonogenic survival and the average number of DNA damage foci per nucleus. Treatment of both cell lines with ^201^Tl in standard incubation media caused a substantial reduction in the surviving fraction compared to non-treated cells. Cells incubated with an equivalent volume of decayed [^201^Tl]TlCl did not show this effect, indicating that the toxicity was solely due to the radioactivity. The average intracellular activity per cell required to achieve 90% reduction in clonogenicity over 90 min was estimated as 0.29 Bq (DU145) and 0.18 Bq (MDA-MB-231). These levels of exposure were accompanied by nuclear DNA damage measurable by the γH2AX assay, which showed a significant increase in the nuclear DNA DSBs caused by ^201^Tl compared to negative controls. Larger standard deviations when measuring nuclear DNA damage could be linked to a heterogeneous uptake of ^201^Tl among cells and/or its unequal subcellular distribution. Factors such as a different cell passage number or different cell cycle phases within the population, might explain large variation in results between experiments. Although nuclear DNA damage might be an important factor leading to a reduction in clonogenic survival, we cannot exclude ^201^Tl impact on other susceptible cellular targets, such us cell membrane or mitochondria, both of which have been linked to the toxicity of other Auger electron-emitting radionuclides [22]. Currently, the precise sub-cellular localization of ^201^Tl in cancer cells remains unknown.

In clonogenic survival studies on MDA-MB-231 cells we used ^201^Tl activity of 8000 kBq/mL in one experiment only as the lower activities used were considered high enough to cause at least 90% reduction in clonogenic survival necessary for the calculations and comparing with the other cell line. In toxicity control figures (Figs. 3b, 4b) showing that various components of decayed ^201^Tl material are not affecting ^201^Tl radiotoxicity, we aimed to use the maximum amount of the radioactive material (and its “cold” equivalent) to be able to observe the potential impact of non-radioactive thallium, if there was one. We therefore limited the number of experiments with higher ^201^Tl activities (4000–8000 kBq/mL) to DU145 (*n* = 3) only or *n* = 1 for MDA-MB-231. This is justified by repeatedly observing no impact of low thallium concentrations on cell toxicity and our efforts to reduce radiation expose caused by the higher doses of gamma radiation.

Based on the subcellular range of Auger electrons (< 1 µm) [11] and their typically low energy, we hypothesized that ^201^Tl has to be internalized to exhibit its radiotoxic effect in cells and that the gamma and X-ray emissions do not contribute significantly to toxicity. To test this hypothesis, we needed a method to modulate ^201^Tl cellular uptake from the medium. Na^+^/K^+^-pump inhibitors ouabain or digoxin, known to be effective in blocking potassium uptake [20], in our experiments caused substantial baseline cytotoxicity in tested cells. However, by varying the K^+^ concentration in the incubation solution between 0 and 25 mM, we were able to achieve a range of ^201^Tl uptake values from 25.9 (0.8 Bq/cell) to 1.6% (0.05 Bq/cell), corresponding to intracellular to extracellular concentration ratios in the range 8:1 to 181:1—a wider range than could be achieved with the cardiac glycosides—without affecting baseline clonogenic survival. When ^201^Tl uptake in cells was substantially suppressed by high K^+^ concentration in the medium, both the loss in clonogenic survival and the increase in DNA DSB caused by ^201^Tl were completely suppressed. This suggests that ^201^Tl needs to be transported inside cells to cause significant radiotoxicity. In the future, if ^201^Tl can be successfully incorporated into a targeted radiopharmaceutical, an optimal dose for the radionuclide administration can be established to achieve an effective therapeutic effect. Furthermore, an impact of low energy gamma and X-ray emission on patients and carers should be investigated to assess feasibility of this treatment. The same principle would apply to any other Auger electron-emitting radiopharmaceutical.

The average of 0.18 Bq/cell for MDA-MB-231 and 0.29 Bq/cell for DU145 of intracellular ^201^Tl required to achieve 90% reduction in clonogenicity over 90 min is equivalent to around 1000–1600 decays per cell. This is less than half the number of decays per cell calculated for ^111^In and ^67^Ga-oxine complexes (3240 and 3600 decays per cell, respectively, over 60-min incubation) required to obtain the same 90% reduction in clonogenicity in DU145 cells [23]. Although these experiments were not conducted under fully identical conditions, this result is consistent with the higher total energy and number of Auger and Coster–Kronig electrons per decay released by ^201^Tl (15.3 keV, 37 electrons) compared to ^111^In and ^67^Ga (6.7 keV, 14.7 electrons and 6.3 keV, 4.7 electrons, respectively) [24]. Because ^201^Tl effluxes quickly from the cell after the incubation period, whereas ^111^In and ^67^Ga do not, the nominal incubation time in the latter cases severely underestimates the actual exposure time, suggesting that ^201^Tl has a much higher radiotoxic potential per Bq.

Our results are consistent with early work by Rao et al. and Kassis et al. [16, 17] addressing potential radiobiological risk associated with diagnostic use of ^201^Tl, showing that decay of intracellular ^201^Tl is toxic, reducing clonogenicity and inducing DNA DSBs. At the same time, ^201^Tl decay presents little hazard to bystander cells that have not taken up the radionuclide. Although we have not compared this potency directly with other Auger electron-emitting radionuclides under consideration as radiotherapeutics, it appears that ^201^Tl is more potent than ^111^In and ^67^Ga, consistent with the respective known number and energy of the emissions. Consequently, ^201^Tl is an attractive candidate radionuclide for further research in targeted Auger electron-emitter therapy. In addressing this goal, it will be necessary to overcome several limitations of the present work. First, while we have shown that ^201^Tl must be internalized to elicit significant radiotoxic effects, it is likely that different subcellular distributions will have greatly differing effects, since the range of the emitted electrons is much smaller than typical cell dimensions. Nothing is known about the subcellular distribution of ^201^Tl in the cell lines investigated here, nor of ^67^Ga and ^111^In in similar studies [23]; indeed, in general the role of subcellular location of radionuclides in determining toxic effects is poorly understood. A radiopharmaceutical containing ^201^Tl that is targeted both to cancer cells and to specific subcellular locations within them might be significantly more potent than [^201^Tl]TlCl. Moreover, ‘trapping’ ^201^Tl inside the cell will slow down its efflux and might further increase its radiotoxic potential. Despite the high potency, actualising therapeutic potential with ^201^Tl is currently more challenging than with other potential Auger electron emitters (^67^Ga, ^111^In, ^125^I etc.) because the chemistry required to incorporate ^201^Tl into targeting molecules is underdeveloped—there are no satisfactory bifunctional chelators for thallium(I) or thallium(III). Here we have used physiological mimicry of potassium to achieve uptake in cells, but this has no specificity for cancer cells and probably has no specific targeting to subcellular structures.

## Conclusion

^201^Tl showed significant radiotoxicity, damaging nuclear DNA and reducing clonogenic survival when internalized in both cancer cell lines used in this study, and had no significant effect if not internalized. These results warrant further investigation of ^201^Tl as a therapeutic radionuclide and the development of chelators to incorporate thallium into targeting molecules for specific delivery of ^201^Tl to cancer cells.

## Supplementary Information

**Additional file 1.** Additional results for In vitro proof of concept studies of radiotoxicity from Auger electron-emitter thallium-201.

## Data Availability

The datasets generated and analysed during the current study are available from the corresponding author on reasonable request.

## Abbreviations

BC: Background count
BSA: Bovine serum albumin
CPM: Counts per minute
DMEM: Dulbecco’s modified eagle medium
PBS: Phosphate buffered saline
RT: Room temperature
