# Supplementary material for: In vitro proof of concept studies of radiotoxicity from Auger electron-emitter thallium-201
Source: EJNMMI Res. 2021 Jul 5;11:63. doi: 10.1186/s13550-021-00802-w (PMC8257813; doi:10.1186/s13550-021-00802-w)
Supplement: Supplementary file 1 — Additional file 1. Additional results for In vitro proof of concept studies of radiotoxicity from Auger electron-emitter thallium-201. [file 13550_2021_802_MOESM1_ESM.docx]

**ELECTRONIC SUPPLEMENTARY MATERIAL**

**Title:** **In vitro proof of concept studies of radiotoxicity from Auger electron-emitter thallium-201 (^201^Tl).**

European Journal of Nuclear Medicine and Molecular Imaging Research

Katarzyna M. Osytek^1^, Philip J. Blower^1^, Ines M. Costa^1^, Gareth Smith^2^, Vincenzo Abbate^3*^ and Samantha Y. A. Terry^1*^

1. King’s College London, School of Biomedical Engineering and Imaging Sciences, London, United Kingdom.

2. Theragnostics Limited, 2 Arlington Square, Bracknell, RG12 1WA, United Kingdom.

3. King’s College London, Department of Analytical, Environmental and Forensic Sciences, London, United Kingdom.

*contributed equally

**Corresponding authors:** Samantha Y. A. Terry, [samantha.terry@kcl.ac.uk](mailto:samantha.terry@kcl.ac.uk); Vincenzo Abbate, [vincenzo.abbate@kcl.ac.uk](mailto:vincenzo.abbate@kcl.ac.uk).

**1. Calibration line for [^201^Tl]TlCl.**

**
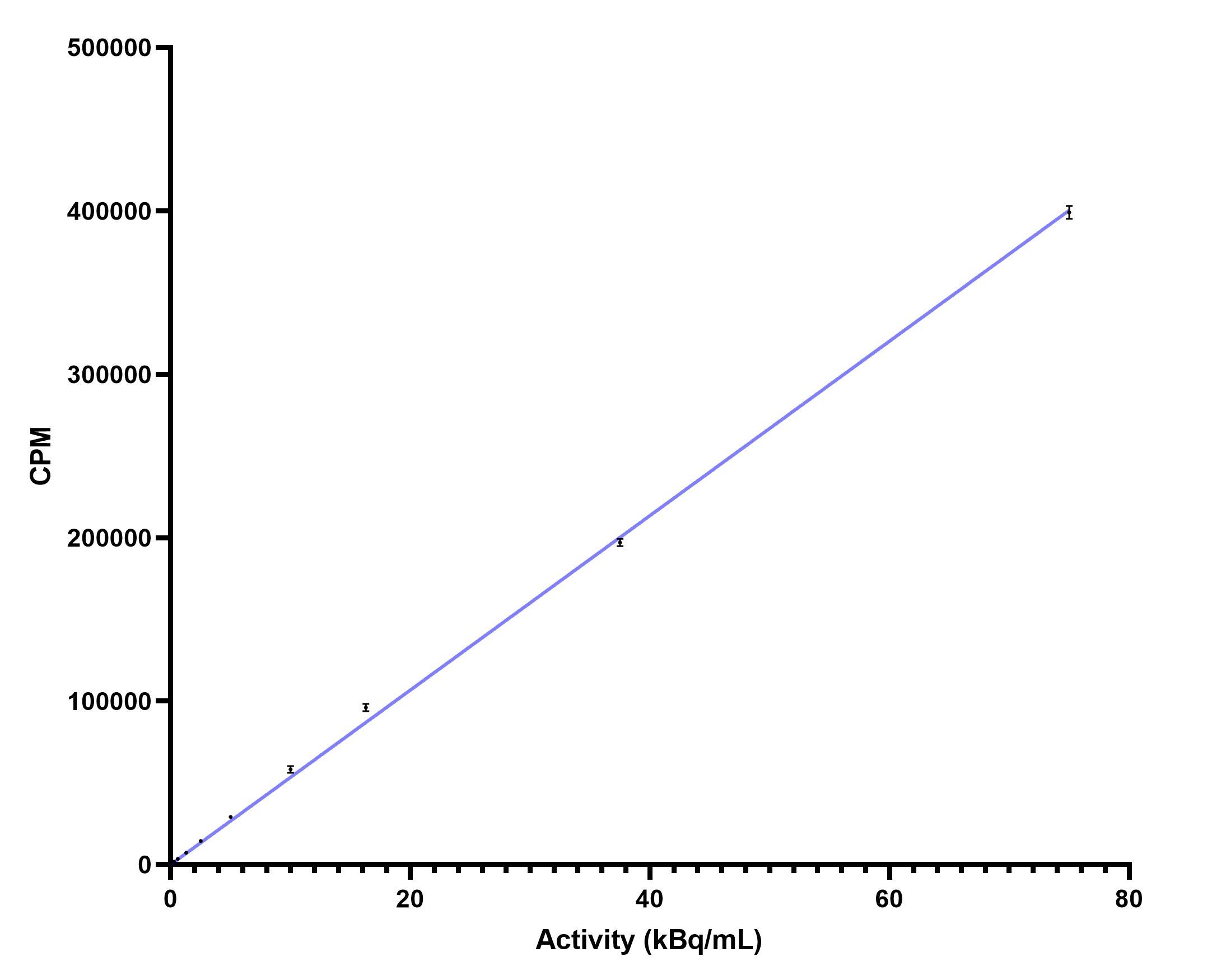
**A series of standards was prepared by diluting 600 kBq/mL of ^201^Tl (measured by a dose calibrator - Capintec) in phosphate buffer solution (PBS) and their radioactivity was monitored by a gamma counter (CompuGamma, CS1282). All activities (0.3 - 600 kBq/mL) were measured in four technical replicates. Measurements with a dead time (DTIME%) value over 20% (marked red) were discarded as unreliable. The gamma counter result (as mean CPM – counts per minute) was plotted against known activity (kBq/mL) and a linear regression line was fitted with R^2^ value of 0.9991. ^201^Tl calibration line was used to convert CPM into ^201^Tl activity required for the efflux assays calculations.

**Fig. 1** Average value of CPM (counts per minute) measured by a gamma counter is plotted against activity checked by a dose calibrator (kBq/mL)

R^2^=0.9991

Y = 5339*X

linear regression

**2. ^203^Tl in [^201^Tl]TlCl supplied (molar activity).**

Natural thallium is a mixture of two stable isotopes: ^205^Tl and ^203^Tl (natural abundance: 70.5% and 29.5%, respectively). ^201^Tl is produced in a cyclotron from a solid target of natural thallium enriched in ^203^Tl isotope [1] (**Fig. 2a**). Inductively Coupled Plasma-Mass Spectrometry (ICP-MS) analysis of a decayed sample of [^201^Tl]TlCl was performed to determine the presence of carrier ^203^Tl and other potential heavy metal contaminants. It confirmed the presence of an isotope with a mass to charge ratio of 203 (**Fig. 2b**) with concentration averaging 0.8 ± 0.4 µmol/L (n=5 x triplicates) (**Table 1**)**,** indicating a molar activity in the range 77 – 240 GBq/μmol at the time of experiments. Neither this level of thallium nor other heavy metals (adventitious lead and the decay product ^203^Hg) were expected to cause toxic effects.

A calibration line for ^203^Tl was prepared (**Fig. 2c**) from a series of natural thallium standards (0.1-200 µg/L) using a thallium solution (100 ppm, Leeman Labs Inc., Teledyne) diluted with 2% HNO_3_ and 0.1% HCl solution (Optima grade, Fisher Scientific). Decayed [^201^Tl]TlCl solution (0.3 mL, in triplicate) was diluted with 2% HNO_3_/0.1% HCl to 5 mL. Quality of the ICP-MS measurements was ensured through repeat measurements of blanks, an external calibrant and an external water reference material from High Purity Standards. The experiment was carried out at argon gas flow of 1 L/min and radiofrequency power of 1600W.

**Fig. 2 a)** Schematic representation of ^201^Tl production and decay process. ^201^Tl is produced in a cyclotron, when the external beam of protons bombards a naturally occurring isotope of ^203^Tl. This nuclear reaction leads to the creation of ^201^Pb (half-life of 9.4 h), which decays by electron capture (EC) to ^201^Tl (half-life of 73 h). ^201^Hg is a stable daughter of ^201^Tl. **b)** Inductively coupled plasma - mass spectrometry (ICP-MS) analysis showing signal intensity versus isotopic mass/charge (m/z) ratio. Oxygen and sodium were identified, as well as a smaller peak for mass to charge ratio (m/z) = 203 indicating ^203^Tl - a cyclotron production process contaminant. Red colour for m/z 203 (^203^Tl) and m/z 205 (^205^Tl) indicate thallium natural abundance. Blue colour for m/z 203 shows non-natural abundance. Lead contamination was within the expected range. The amount of the isotope with m/z ratio of 201 (^201^Hg) was below the detection limit for this method. **c)** A calibration line showing concentrations of prepared TlNO_3_ standards (µg/L) plotted against ^203^Tl signal intensity in counts per second (CPS). Linear regression was fitted with R^2^ value of 0.9999


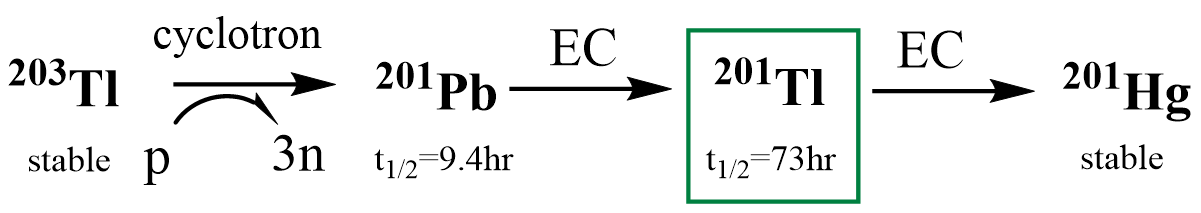


**a**

**b**


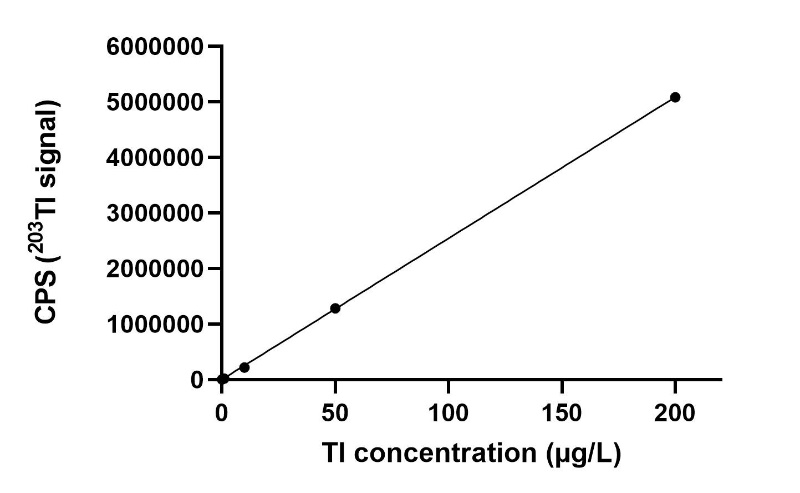


R^2^=0.9999

Y = 25425*X

linear regression

**c**

**3. ^201^Tl kinetics in DU145 cells and MDA-MB-231 cells.**


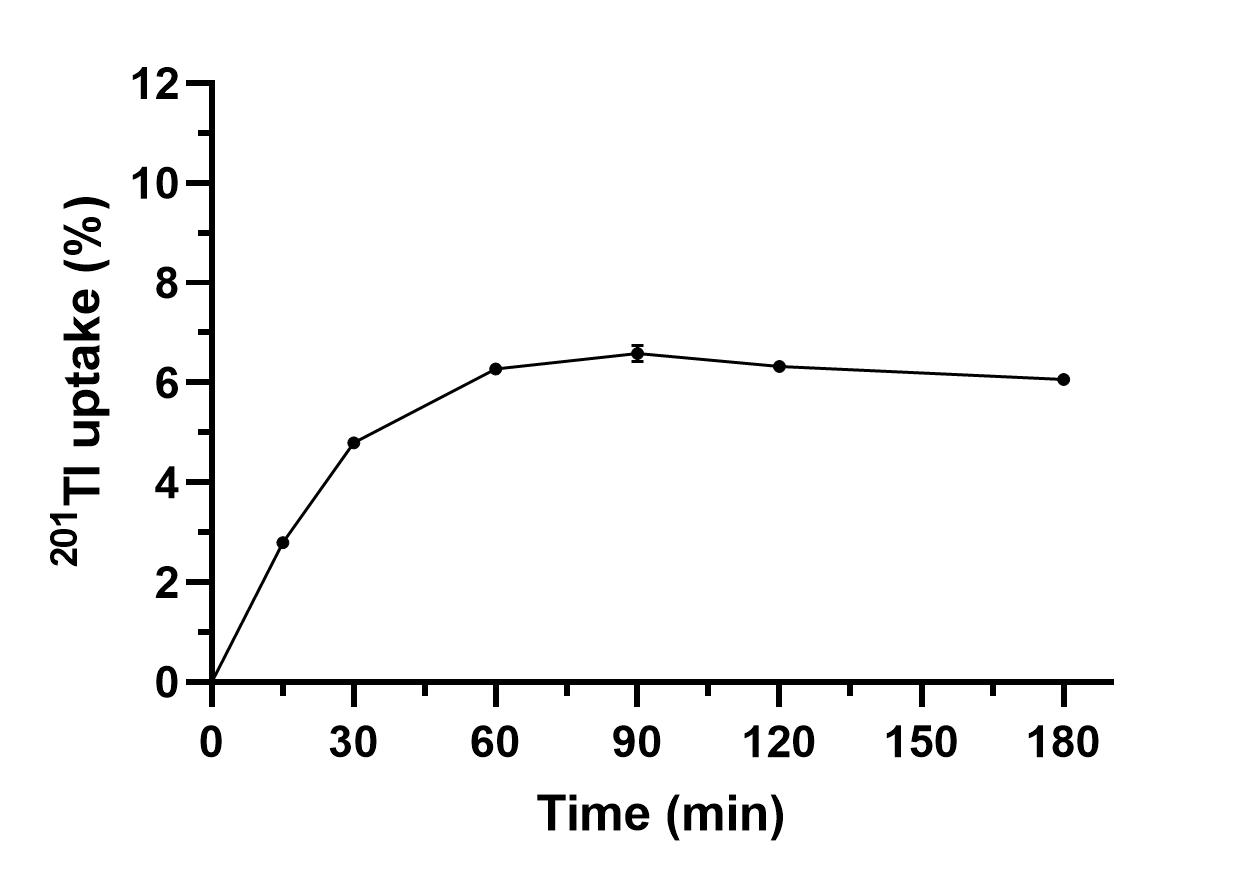


**Fig. 3 ^201^Tl kinetics. a)** Uptake of ^201^Tl in MDA-MD-231 cells (n=1) after incubation with 200 kBq/mL [^201^Tl]TlCl in medium. These results were used to determine the best incubation time for subsequent radiobiological experiments. **b)** Efflux of ^201^Tl in MDA-MD-231 (n=1) cells. Plateau uptake from the preceding uptake assay is defined as 100% at time 0. Radioactive medium was replaced with non-radioactive and ^201^Tl activity was measured over time. **c)** ^201^Tl intracellular activity (Bq) per single cell in DU145 cells (n=2) and MDA-MD-231 cells (n=1) after radioactive medium was replaced repeatedly with non-radioactive medium over time, performed on the same cells in quadruplicates. Intracellular activity per cell at 0 min is the activity bound in a preceding uptake assay; presented as mean ± SD, done in triplicates; exponential regression line was fitted in b. Some error bars are smaller than the data symbols and hence not visible

**a**

**b**


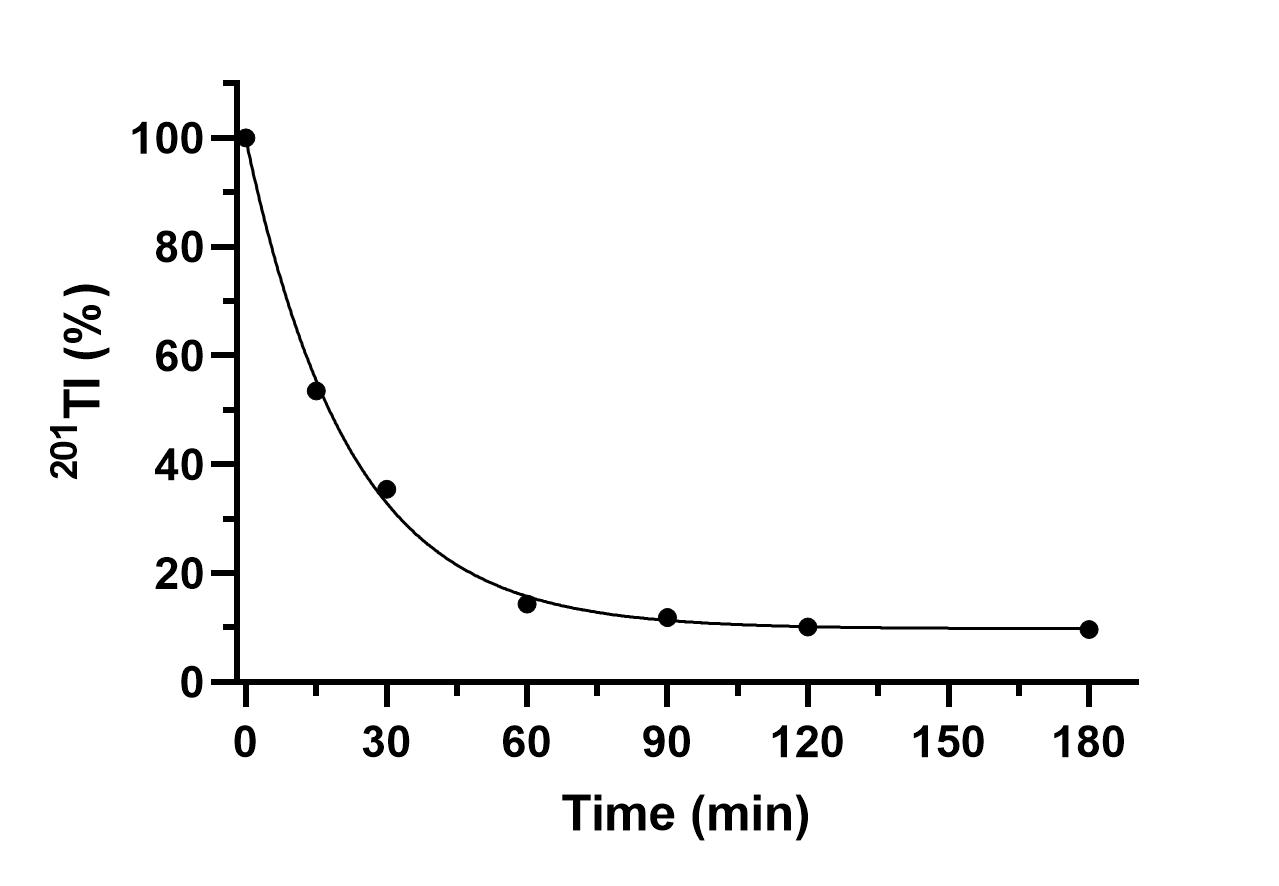


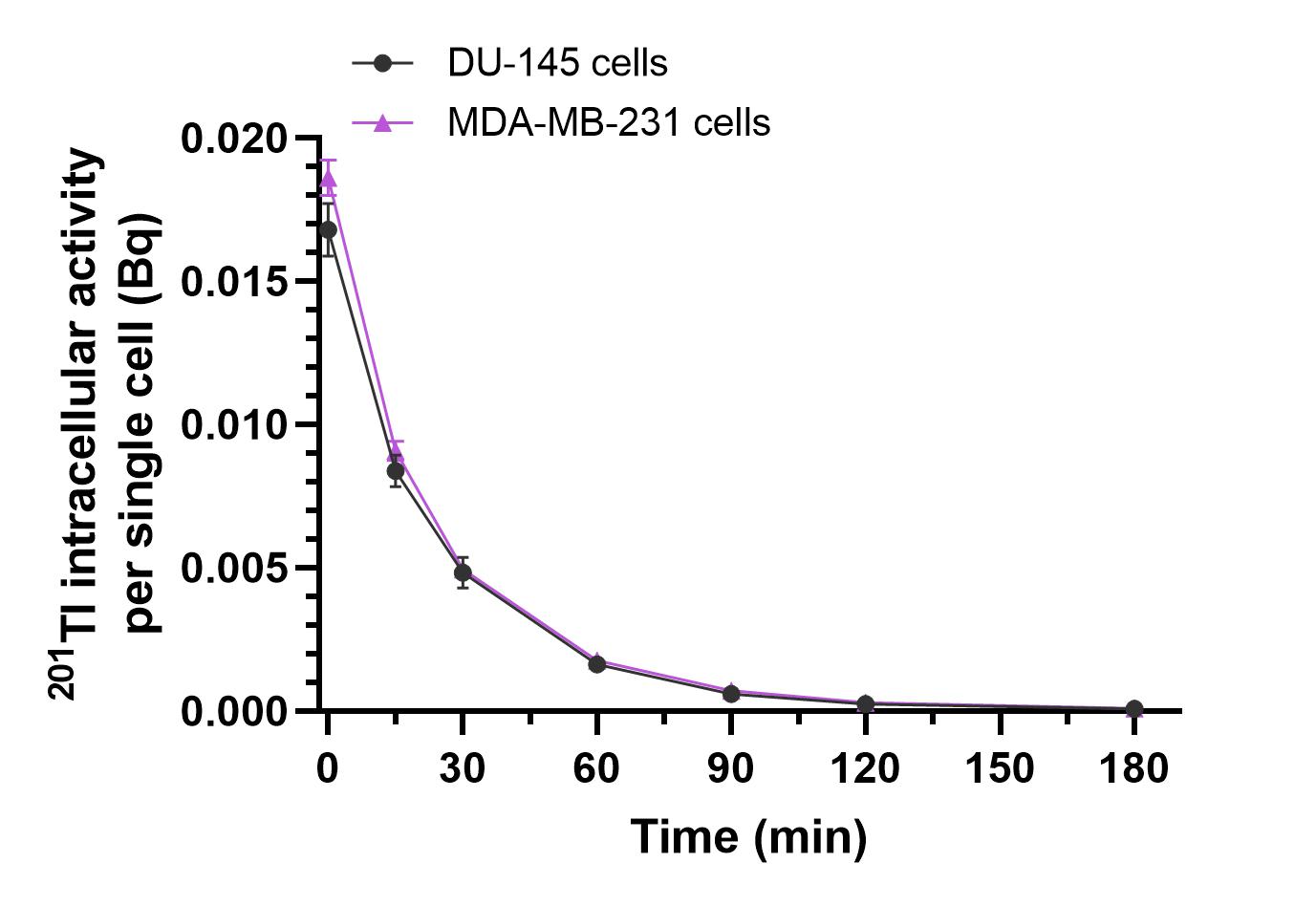


**c**

**4. Impact of cardiac glycosides on ^201^Tl uptake and cell survival.**

To compare the radiotoxicity caused by ^201^Tl present in the extracellular medium and those inside cells, ^201^Tl uptake needed to be substantially reduced. Cardiac glycosides (ouabain, digoxin) are known to efficiently block the Na^+^/K^+^-ATPase pump [2] and were found to decrease the amount of internalised ^201^Tl in DU145 cells by 59.5 - 83.9% (**Fig. 4a**), but they also caused a 56.8 - 98.9% reduction in baseline clonogenic survival (**Fig. 4b**), therefore alternative methods to control ^201^Tl uptake were sought**.**  Digoxin (0.5 mg/2 mL solution) for intravenous injection (Aspen) was diluted with 0.9% NaCl to the required concentration. Ouabain solutions were prepared by dissolving ouabain octahydrate in 0.9% NaCl.


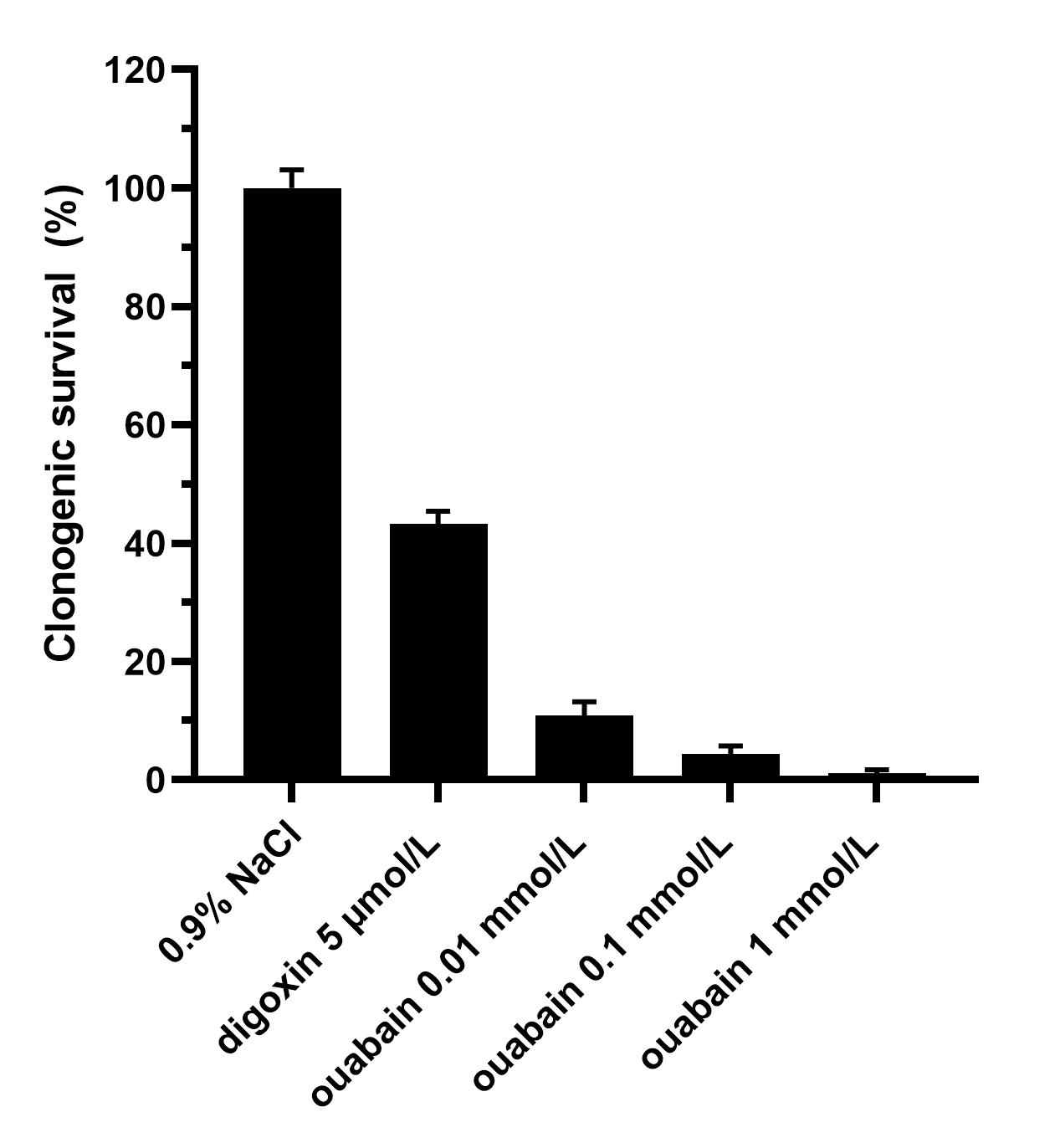

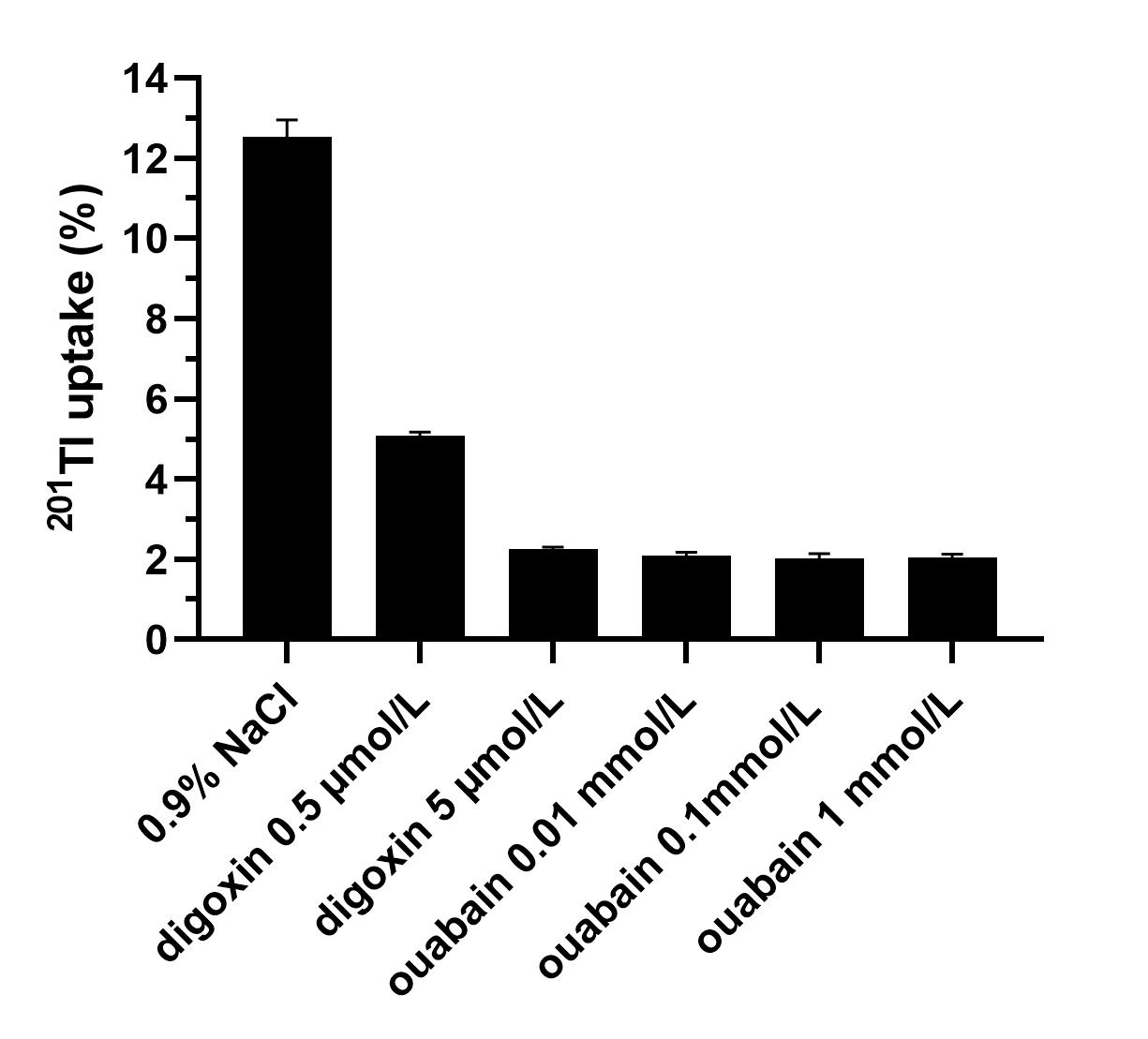


**Fig. 4** **a)** ^201^Tl uptake in medium measured in DU145 cells after 90 min incubation with 80 kBq/mL of [^201^Tl]TlCl and different concentrations of digoxin (0.5 - 5 μmol/L) or ouabain (0.01 – 1 mmol/L in 0.9% NaCl). **b)** Clonogenic toxicity of cardiac glycosides expressed as surviving percentage measured in DU145 cells after 90 min incubation with 5 µmol/L of digoxin or 0.01 - 1 mmol/L of ouabain in medium. Cells were not exposed to ^201^Tl in these experiments. **c)** ^201^Tl uptake in DU145 cells after 90 min incubation with 80 kBq/mL [^201^Tl]TlCl and different concentrations of KCl in PBS without K^+^ (n=1), showing that KCl solution can efficiently modulate ^201^Tl uptake. Data are presented as mean ± SD, n=1, experiments were performed in triplicates

**b**

**a**


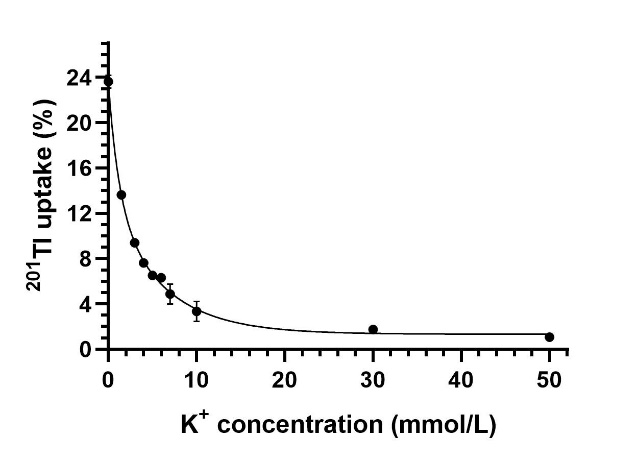


**c**

**5.** **^201^Tl** **radiotoxicity – nuclear DNA damage.**


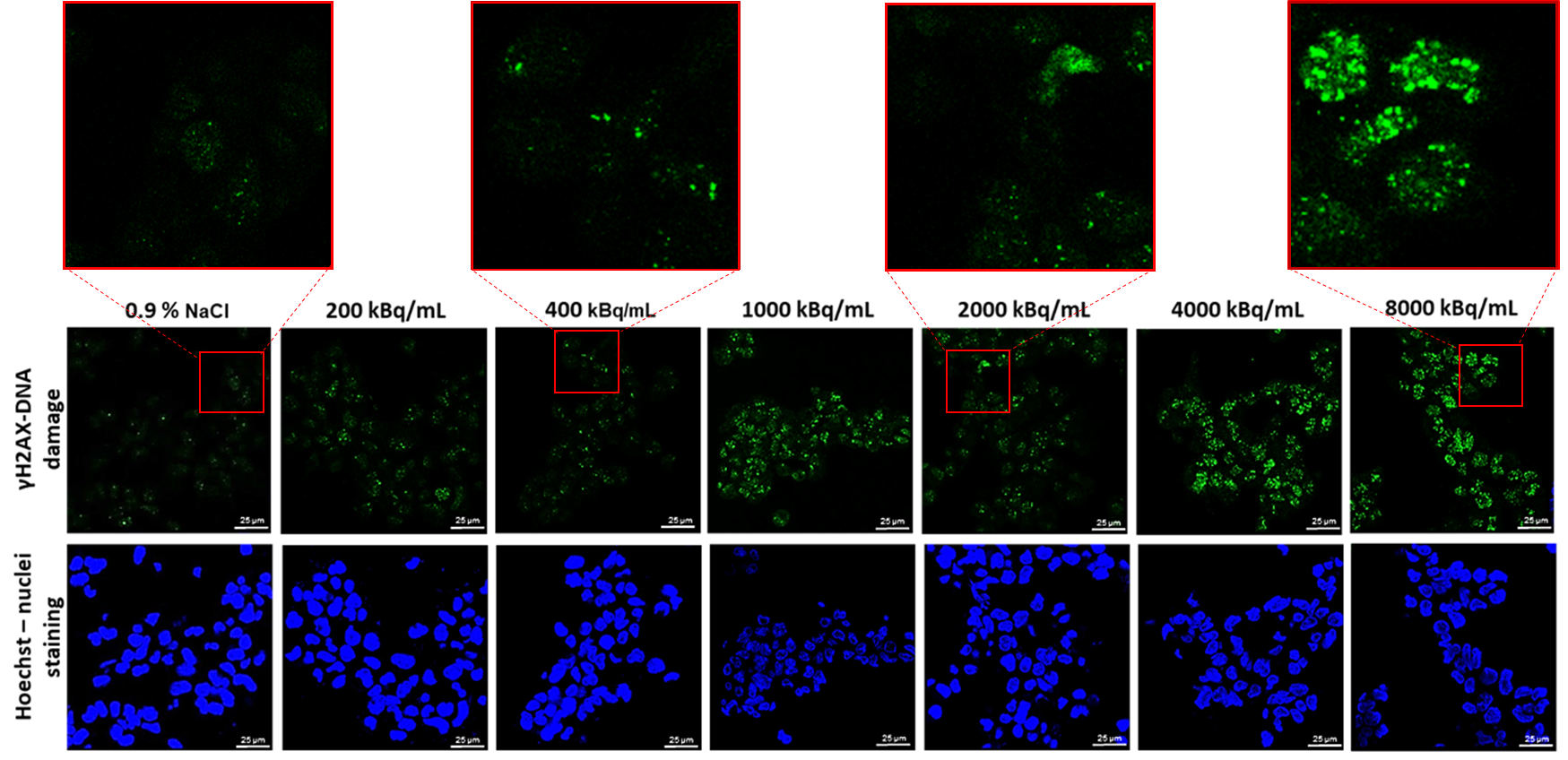


**a**

**
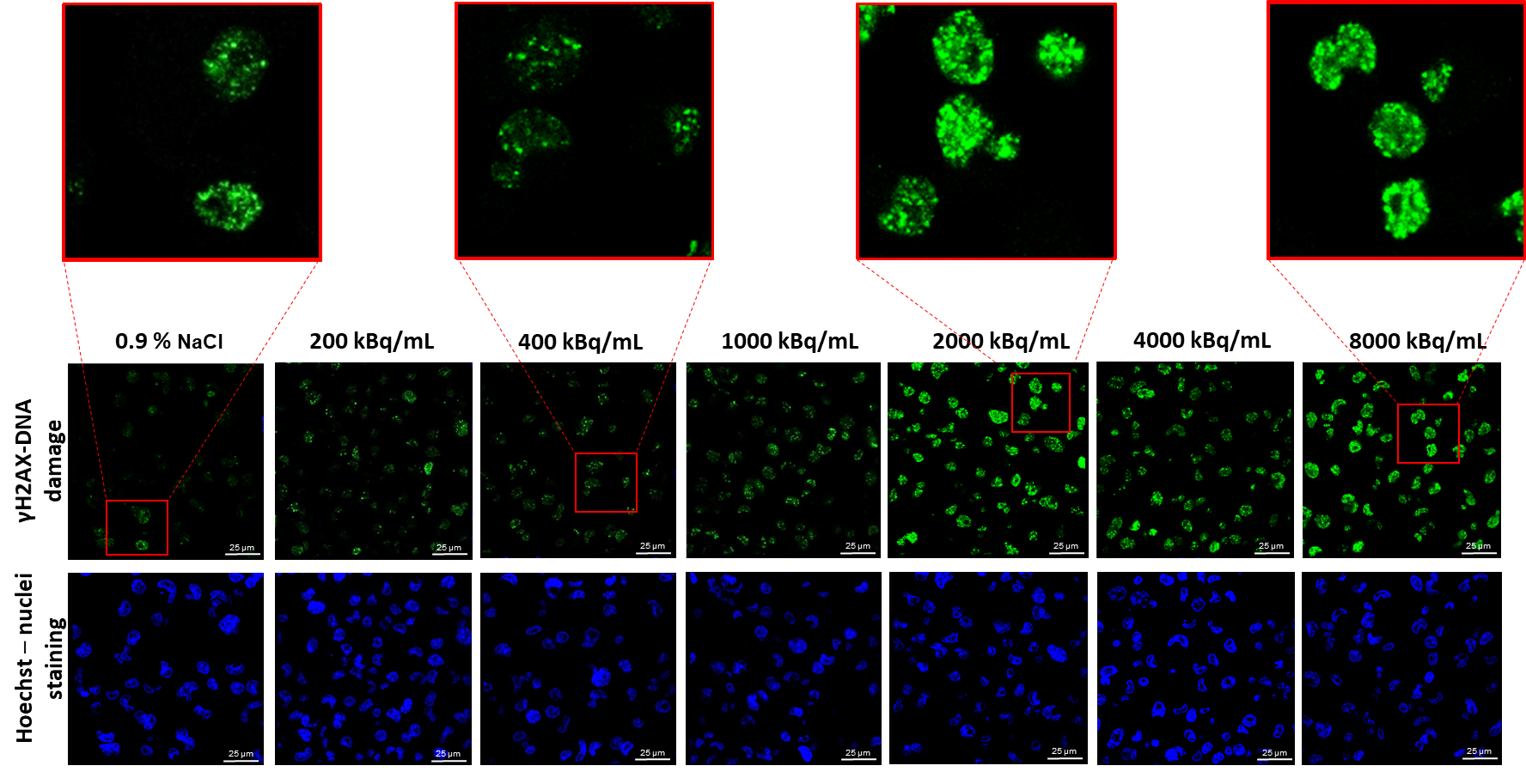
**

**b**

**Fig. 5** Exemplar confocal microscopy images (100x) of **a)** DU145 cells and **b)** MDA-MB-231 cells incubated for 90 min with 0.9% NaCl (negative control) or 200 – 8,000 kBq/mL [^201^Tl]TlCl, followed by immunofluorescence staining for γH2AX (green). Nuclear DNA is stained with Hoechst (blue); minimum 25 cells visible, Z-stack size was kept constant throughout all experiments; for the AlexaFluor green fluorescence: smart gain (SG) and smart offset (SO) were kept constant for all conditions in the experiment. Scale bar – 25 μm

**TABLES:**

**Table** **1.** **Inductively coupled plasma - mass spectrometry (ICP-MS) quantification analysis**

| **Batch** | **Average concentration (μmol/L)** | **SD** |
| --- | --- | --- |
| **1** | 0.75 | 0.02 |
| **2** | 1.14 | 0.03 |
| **3** | 0.45 | 0.003 |
| **4** | 0.35 | 0.06 |
| **5** | 1.29 | 0.02 |
| **Average** | 0.80 | 0.41 |

Inductively coupled plasma - mass spectrometry (ICP-MS) quantification analysis of five different batches of decayed [^201^Tl]TlCl solution showing concentrations (μmol/L) for isotopic mass/charge (m/z) ratio of 203, indicating the concentration of ^203^Tl - a cyclotron production process contaminant. [^201^Tl]TlCl was obtained as 0.9% NaCl solution; 360 - 560 MBq/5.8 mL

| **Batch** | **Average concentration (μmol/L)** | **SD** |
| --- | --- | --- |
| **1** | 0.75 | 0.02 |
| **2** | 1.14 | 0.03 |
| **3** | 0.45 | 0.003 |
| **4** | 0.35 | 0.06 |
|  |  |  |
| **5** | 1.29 | 0.02 |
| **Average** | 0.80 | 0.41 |
|  |  |  |

Inductively coupled plasma - mass spectrometry (ICP-MS) quantification analysis of five different batches of decayed [^201^Tl]TlCl solution showing concentrations (μmol/L) for isotopic mass/charge (m/z) ratio of 203, indicating the concentration of ^203^Tl - a cyclotron production process contaminant. [^201^Tl]TlCl was obtained as 0.9% NaCl solution; 360 - 560 MBq/5.8 mL

**Table** **2. Average ratio of intracellular to extracellular concentrations of ^201^Tl and K^+^**

**a**

| **Time of incubation (min) with 200 kBq/mL of ^201^Tl in RPMI medium** | **Average ^201^Tl concentration (kBq/µl) ± SD inside DU145 cells, n=3** | **Average ^201^Tl concentration (kBq/µl) ± SD in external medium, n=3** | **Internal to external ^201^Tl concentration ratio, n=3** |
| --- | --- | --- | --- |
| 30 | 7.59 ± 3.03 | 0.19 ± 0.01 | 40 |
| 60 | 8.86 ± 3.32 | 0.19 ± 0.01 | 47 |
| 90 | 9.16 ± 3.55 | 0.19 ± 0.01 | 49 |
| 120 | 9.09 ± 3.40 | 0.19 ± 0.01 | 49 |
| 180 | 9.89 ± 3.25 | 0.19 ± 0.01 | 53 |

**a**

| **Time of incubation (min) with 200 kBq/mL of ^201^Tl in RPMI medium** | **Average ^201^Tl concentration (kBq/µl) ± SD inside DU145 cells, n=3** | **Average ^201^Tl concentration (kBq/µl) ± SD in external medium, n=3** | **Internal to external ^201^Tl concentration ratio, n=3** |
| --- | --- | --- | --- |
| 30 | 7.59 ± 3.03 | 0.19 ± 0.01 | 40 |
| 60 | 8.86 ± 3.32 | 0.19 ± 0.01 | 47 |
| 90 | 9.16 ± 3.55 | 0.19 ± 0.01 | 49 |
| 120 | 9.09 ± 3.40 | 0.19 ± 0.01 | 49 |
| 180 | 9.89 ± 3.25 | 0.19 ± 0.01 | 53 |

| **Activity of ^201^Tl (kBq/mL) added (90 min incubation) in RPMI medium** | **Average ^201^Tl concentration (kBq/µl) ± SD inside DU145 cells, n=3** | **Average ^201^Tl concentration (kBq/µl) ± SD in external medium, n=3** | **Internal to external ^201^Tl concentration ratio, n=3** |
| --- | --- | --- | --- |
| 200 | 9.61 ± 2.87 | 0.18 ± 0.01 | 53 |
| 400 | 18.18 ± 5.94 | 0.36 ± 0.02 | 50 |
| 1000 | 43.43 ± 13.92 | 0.91 ± 0.04 | 48 |
| 2000 | 82.29 ± 26.16 | 1.83 ± 0.07 | 45 |
| 4000 | 165.32 ± 53.13 | 3.67 ± 0.15 | 45 |
| 8000 | 324.91 ± 114.75 | 7.35 ± 0.31 | 44 |

**b**

| **Concentration (mmol/L) of K^+^ in the incubation solution (90 min incubation with 4000 kBq/mL ^201^Tl)** | **Average ^201^Tl concentration (kBq/µl) ± SD inside DU145 cells, n=3** | **Average ^201^Tl concentration (kBq/µl) ± SD in external solution, n=3** | **Internal to external ^201^Tl concentration ratio, n=3** |
| --- | --- | --- | --- |
| 0 | 537.75 ± 51.42 | 2.96 ± 0.07 | 181 |
| 15 | 45.62 ± 7.74 | 3.91 ± 0.01 | 12 |
| 25 | 32.76 ± 11.07 | 3.94 ± 0.02 | 8 |

**c**

Average ratio of intracellular to extracellular concentrations of ^201^Tl and K^+^ **a)** DU145 cells incubated for 30 – 180 min with 200 kBq/mL of [^201^Tl]TlCl in RPMI medium. **b)** DU145 incubated with various ^201^Tl activities ranging between 200 and 8000 kBq/mL for 90 min in RPMI medium. **c)** DU145 incubated for 90 min with 4000kBq/mL of [^201^Tl]TlCl in PBS without K. The intracellular to extracellular concentrations ratio of ^201^Tl was calculated using the uptake experiments results (n=3) in DU145 cells. Experimental DU145 intracellular volume, measured by diffusion NMR spectroscopy [3] was taken from literature as 1.6 pL.

**REFERENCES**

1. Sattari I, Aslani G, Dehghan MK, Shirazi B, Shafie M, Shadanpour N, et al. Dependence of quality of thallium-201 on irradiation data. Iran J Radiat Res. 2003;1:51–4.

2. Ogawa H, Cornelius F, Hirata A, Toyoshima C. Sequential substitution of K^+^ bound to Na^+^,K^+^-ATPase visualized by X-ray crystallography. Nat Commun. 2015;6:8004.

3. Pilatus U, Shim H, Artemov D, Davis D, van Zijl PCM, Glickson JD. Intracellular volume and apparent diffusion constants of perfused cancer cell cultures, as measured by NMR. Magn Reson Med. 1997;37:825–32.
